# Hyperinsulinemia: an early biomarker of metabolic dysfunction

**DOI:** 10.3389/fcdhc.2023.1159664

**Published:** 2023-05-02

**Authors:** Rama A. Vaidya, Sharvari Desai, Panchali Moitra, Sheryl Salis, Shubhada Agashe, Rekha Battalwar, Anushree Mehta, Jagmeet Madan, Soumik Kalita, Shobha A. Udipi, Ashok B. Vaidya

**Affiliations:** ^1^ Kasturba Health Society- Medical Research Center, Mumbai, India; ^2^ Sir Vithaldas Thackersey College of Home Science (Autonomous), Shreemathi Nathibai Damodar Thackersey Women’s University, Mumbai, India; ^3^ Nurture Health Solutions, Mumbai, India; ^4^ Clinical and Endocrine Laboratory, Kasturba Health Society Medical Research Centre, Mumbai, India; ^5^ FamPhy: Gurgaon, Haryana, India

**Keywords:** hyperinsulinemia, HbA1c, prediabetes, metabolic syndrome, insulin resistance

## Abstract

**Introduction:**

Hyperinsulinemia in the absence of impaired glucose tolerance and normal HbA1c is considered indicative of pre-diabetes. Very few Indian studies have focused on hyperinsulinemia particularly in young adults. The present study aimed to determine whether hyperinsulinemia may be present despite HbA1c being normal.

**Methods:**

This was a cross–sectional study conducted on adolescents and young adults aged 16-25 years living in Mumbai, India. The participants attended various academic institutions and were those who underwent screening as the first step of a clinical trial for studying the efficacy of almond intake in prediabetes.

**Results:**

Among this young population (n=1313), 4.2% (n=55) of the participants were found to be prediabetic (ADA criteria) and 19.7% of them had HbA1c levels between 5.7%-6.4%. However, almost, 30.5% had hyperinsulinemia inspite of normal blood glucose levels and normal HbA1c. Among those with HbA1c<5.7 (n=533), 10.5% (n=56) participants had fasting insulin>15 mIU/L and a higher percentage (39.4%, n=260) had stimulated insulin above 80 mIU/L. These participants had higher mean anthropometric markers than those with normal fasting and/or stimulated insulin.

**Conclusion:**

Hyperinsulinaemia in the absence of impaired glucose tolerance and normal HbA1c may provide a much earlier indicator of detection for risk of metabolic disease and progression to metabolic syndrome and diabetes mellitus.

## 1 Introduction

Type 2 Diabetes(T2D) is a metabolic, endocrine disorder, that is reaching epidemic proportions globally. However, the increase in South East Asia (68%) is alarming and India ranks second in the world for the number of adults (20-79 years of age) with diabetes and will continue to have the second highest number (174.4 million) by 2045 (International Diabetes Federation, 2021) (1).

The ICMR-INDIAB study, indicated that the overall prevalence of T2D in 15 Indian states was 7·3%, and that of prediabetes was 10.3% in individuals above 20 years in 14 Indian states (2). The 5^th^ National and Family Health Survey (2019–2021) (3), reported that 16.8% of the adult males and 14.6% of the adult females were diabetic, based on any one of the three criteria: high blood sugar levels (141-160 mg/dl) or very high blood sugar levels (>160mg/dl) or if the person was taking medication to control the blood sugar level. However, glycosylated haemoglobin (HbA1c) was not measured, nor was prediabetes addressed. Impaired glucose tolerance (IGT) and impaired fasting glucose (IFG) are important to stave off the progression to T2D (IDF, 2021) (1). In persons with IGT and IFG, blood glucose levels are generally below the diagnostic thresholds for diabetes. However, they indicate that the risk of developing T2D (4–6) and cardiovascular disease (CVD) (7, 8) is higher. The estimated cumulative incidence of progression to type 2 diabetes, five year. post the diagnosis of IGT is about 26% and 50% in case of IFG (8, 9). Therefore, early detection provides a sizeable and long enough window of opportunity to prevent development of this metabolic disorder. In India, an estimated 39.4 million adults or 53.4 percent (20-79 years) are reported to be undiagnosed with diabetes (IDF, 2021) (1). Age and weight influence the progression from IGT and IFG to type 2 diabetes. Such huge numbers undoubtedly place a great burden on the health care system and compromise the health and quality of life of an individual and the family, as well as having an economic impact in terms of increased health care costs.

Prediabetes is commonly an asymptomatic condition that can exist for years before diabetes is evident. Elevation of blood sugar is a continuum and prediabetes is not a benign condition, since it is associated with increased risk of type 2 DM and CVD as well as all-cause mortality (10, 11). Diagnosis of both diabetes and prediabetes are based on the oral glucose tolerance test (OGTT) that requires measurements of glucose concentrations in fasting state and at two hours post-stimulation with 75grams of glucose and/or the measurement of HbA1c (10, 12). The American Diabetes Association (ADA, 2022) has recommended the cut-off value of 140-199 mg/dL for IGT and a lower cut-off value of 100-125 mg/dL for IFG. Additionally, it has recommended that HbA1c between 5.7% to 6.4% can be used to diagnose prediabetes and an HbA1c value ≥6.5% can be considered as a cut off for diabetes in asymptomatic patients (13, 14). Mohan et al. (2010) reported that in Asian Indians, HbA1c of ≥6.0% accurately identifies diabetes. A value of 5.6% optimally identified IGT or IFG, but it was less than 70% accurate with greater chances of missing out at-risk individuals (15). Several investigators (16–19) have observed racial and ethnic variations in HbA1c values for the same degree of hyperglycaemia affecting its utilization as a modality for diagnosing diabetes (19–21).

Insulin resistance is well recognized as a very good predictor for developing type 2 DM in the future (22, 23). Hypersecretion of insulin and hyperinsulinemia could occur prior to insulin resistance and contribute not only to T2D but also the risk of CVD (23, 24). In insulin resistance the characteristically higher circulating insulin levels are required to achieve an integrated glucose-lowering response. This burdens the endocrine pancreas and the β-cell decompensation that occurs, pave the way for development of overt type 2 DM.

In 1975, Kraft highlighted that hyperinsulinemia manifests itself much before hyperglycemia and therefore, diabetes ‘*in situ*’ can be identified at an earlier stage, much before markers like HbA1c indicate dysglycemia (25). Therefore, it is important to identify people who are at risk of developing T2D, particularly because even before diagnosis, vascular complications could have set in. Crofts et al. (2016) proposed that hyperinsulinemia should be considered independently to insulin resistance, in view of its role in contributing as a direct or indirect factor to metabolic diseases (26).

Indians are prone to T2D, probably attributable to compromised metabolic capacity (27) and screening Indians <30 years for NCDs is being recommended (28). Between 2017 and 2019 we screened 1313 young adults as part of an intervention study. During this, wherein we observed that a considerable number of participants had high insulin levels while their HbA1c levels were normal, and very few were diabetic or prediabetic by ADA, 2017 criteria (29). Therefore, we considered it worthwhile to examine the fasting and 2-hour stimulated glucose and insulin levels vis-à-vis their HbA1c levels.

## 2 Materials and methods

### 2.1 Study design and sample selection

This cross –sectional study was conducted on adolescents and young adults (16-25 years of age) in Mumbai, India. The participants were young adult males and females who were attending academic institutions in Mumbai city and underwent screening for a clinical trial for studying the efficacy of almond intake in prediabetes (30). Twenty- four academic institutions in Mumbai city were contacted and the study objectives and protocol were explained to the administration/authorities. Eleven of these institutes permitted to recruit their students for the study. A total of 1313 young adults volunteered to undergo screening. Among them, 34% (n=446) were 16 to 18 years old, the remaining 66.0% (n=867) were aged 19 to 25 years. All participants were enrolled after obtaining informed written consent and for those between 16-18 years informed written parental consent was obtained. Exclusion criteria included presence of any known chronic disease, known history of food allergies with nuts, on prescribed medications like steroids, state of pregnancy and/or lactation.

#### 2.1.1 Ethical considerations

The study was approved by the Intersystem Biomedical Ethics Committee, Mumbai, India (ISBEC version 2 dated 12^th^ Aug, 2017) and conducted according to Good Clinical Practices and the Declaration of Helsinki.

### 2.2 Measurements

Each participant was examined by a physician to assess the general health status. Weight, height, waist circumference and hip circumference were measured. Body composition was measured using the TANITA body composition analyser (Model MC 780 MA). Each measurement was taken thrice and the average was calculated.

Weight- Participants were weighed using a calibrated digital weighing scale (Equinox, Model EB6171, accuracy 0.1kg). It was ensured that they were wearing light clothing and no footwear at the time of measurement. The scale was zeroed before every measurement.

Height- It was measured using a stadiometer (accuracy of 0.1cm). Subjects were asked to remove their footwear, stand with their feet together, knees straight and chin parallel to the ground. Care was taken that the back of the head (occipital lobe), shoulder blades, buttocks and heels were in contact with the stadiometer surface.

Body Mass Index (BMI) was calculated as weight/height^2^ (kg/m^2^) and participants were classified as underweight, normal, overweight or obese based on the WHO criteria for Asians (2004).

Waist circumference (WC) and hip circumference (HC) were measured with a calibrated, non-extensible, flexible measuring tape. WC was measured at a level midway between the bottom of the rib cage and superior margin of iliac crests during inspiration and hip circumference at the maximal diameter of the buttocks. Waist-to-hip ratio (WHR) and waist-to- height ratio (WHtR) were calculated. WHtR≥0.50 was considered as the optimal cut-off (31).

### 2.3 Biological samples, collection, storage and biochemical measurements

Participants reported to the laboratory after an overnight fast of at least 12 hours. Venous blood (10ml) was collected in fasting state and four ml of blood was collected 2 hrs post 75 gms glucose by a trained phlebotomist. Two mL of fasting blood sample was immediately transferred to a BD vacutainer (spray-coated K2EDTA Tubes) for complete blood count (CBC) and HbA1c, two ml of fasting and post glucose blood sample was immediately transferred to a BD vacutainer (spray-coated sodium fluoride tubes) for estimation of plasma glucose levels. The remaining six ml of fasting blood and 2 ml of post glucose blood were transferred into plain BD vacutainer for separation of serum. The vacutainers were transported on ice to the Institute’s laboratory. Fluoride and plain vacutainers were centrifuged, fluoride plasma was processed for estimation of plasma glucose levels and serum was processed for serum insulin levels. The remaining fasting serum was divided into aliquots and stored at -70^0^C until further analyses.

Glucose tolerance test (fasting and 2-h post 75-g glucose administration) was conducted for all 1313 participants. Glucose was measured by the GOD POD method (Accurex Biomedical Pvt Ltd), insulin was measured by radioimmunoassay using a Beckman Coulter Counter. HbA1c was measured using Nycocard reader (Alere Technologies, Norway) for 667 participants of the 1313 participants.

Participants with fasting glucose levels between 100-125mg/dL (5.6-6.9 mmol/L) and 2-hour post- glucose value 140-199 mg/dL (7.8-11.0 mmol/L) were designated as prediabetic (29). Hyperinsulinemia was defined as fasting hyperinsulinemia (≥15 mIU/ml) or glucose challenge hyperinsulinemia (≥ 80m IU/ml) (32, 33).

### 2.4 Statistics

Descriptive data of participants are reported as mean ± SD and 95% confidence interval (CI) for continuous variables. Student’s 2-tailed t-test and Pearson’s Chi Square analysis were applied using STATA (14.2). A p-value <0.05 was set to determine statistically significant differences.

## 3 Results

The mean age of the study sample (n=1313) was 19.6 ± 2.1 years. History of diabetes among first degree relatives were recorded in 22.5% of the participants and that of second degree relative was 43.7% of the participants. The overlap in first- and second-degree relatives having diabetes was 12.4% (**Table 1**). A high percentage of the participants were either overweight (15.2%, n=200) or obese (20.5%, n=269) and 23.3% (n=306) were underweight. Based on the ADA criteria, when we considered fasting glucose levels between 100-125mg/dL (5.6-6.9 mmol/L) and 2-hour post-glucose value of 140-199 mg/dL (7.8- 11.0 mmol/L), 4.2% (n=55) of the participants out of 1313 were found to be prediabetic. When HbA1c was used for diagnosis (13), 19.7% of the participants out of 667 were found to be prediabetic with HbA1c levels between 5.7%-6.4% and three participants had HbA1c levels ≥6.5%. We have also used circulating levels of insulin, fasting (≥ 15 mIU/ml) and 2 hours glucose stimulated levels (≥ 80m IU/ml) be considered as prediabetic. A fairly high percentage of the young adults 30.5% (n=400) were hyperinsulinemic (**Table 1**).

**Table 1:** Characteristics of 1313 study participants.

Characteristics	n (%)	Mean ±SD Median 95%CI Overall (n=1313)
Gender		
Males Females	457 (34.8) 856 (65.2)	
Family history of diabetes (n=1313)		
First degree family member (parents/siblings) Second degree family member (grandparents/ uncle/aunts) Both first- and second-degree family members No family member with diabetes	132(10.1) 408 (31.1) 163 (12.4) 610 (46.4)	
Markers of Glucose Tolerance (n=1313)		
Normal blood glucose and insulin levels Prediabetes (ADA criteria) Hyperinsulinemia but normal Blood Sugars Diabetes Fasting Blood Glucose ≥100mg/dl 2 - hour post Glucose≥140mg/dl Fasting Insulin ≥15 mIU/ml Stimulated Insulin ≥80 mIU/ml Both Fasting and Stimulated Insulin above normal	855(65.1) 55(4.2) 400 (30.5) 3(0.23) 28(2.1) 31(2.4) 118(9.0) 400(30.5) 82(1.37)	82.7±11.5 (82.0, 81.8-83.6) 96.2±26.0 (93.9, 94.2-98.1) 9.1±4.7 (8.0, 8.7-9.4) 80.0±61.8 (62.0, 75.3-84.7)
HbA1c (n=667)		5.45±0.5 (5.0, 5.4-5.5)
HbA1c (≤5.7%) HbA1c (5.75-6.4%) HbA1c (≥6.5%)	533(79.6) 131(19.9) 3(0.4)	
**Body Mass Index (n=1313)** Underweight	306(23.3)	
Normal weight Overweight Obese	538(41.0) 200(15.2) 269(20.5)	
Central adiposity measures (n=1313)		
Waist Circumference >80 cm in Females >90 cm in Males Waist to height ratio >0.5	108/856 (12.6) 38/457 (8.31) 280(21.3)	

Mean values for all anthropometric indicators as well as the percentages of subjects who were overweight or obese, those with higher amount of body fat or visceral fat were calculated (**Table 2**). For the prediabetic participants with HbA1c between 5.7% and 6.5%, percent body fat was significantly higher than the subjects with normal HbA1c. The mean values for BMI, waist circumference, WHR, waist to height ratio, total percent body fat, visceral fat and muscle mass did not differ significantly.

**Table 2:** Comparison of anthropometric indicators in participants with normal HbA1c (<5.7) and prediabetics (HbA1c: 5.7- 6.4).

Anthropometric Measurements		HbA1c <5.7 (n=533) n (%)	HbA1c 5.7-6.4 (n=131) n (%)	t value	P
Mean ±SD					
Body mass index (BMI) (kg/m^2^)		22.4±4.3	23.1±5.4	-1.621	0.106
Waist circumference (WC) (cm)		71.1±9.9	71.6±10.8	-0.483	0.630
Waist:hip ratio (WHR)		0.76±0.06	0.76±0.06	0.865	0.388
Waist: height ratio (WHtR)		0.45±0.06	0.45±0.07	-1.230	0.219
Percent Body Fat (%)		28.2±8.2	30.1±8.4	-2.443	0.015
Visceral Fat		4.2±2.9	4.5±3.1	0.646	0.345
Muscle Mass (kg)		37.7±8.0	36.9±7.2	1.109	0.268
Number, (%) Subjects with Normal and Higher Anthropometric Indices				Chi sq	P
BMI (kg/m^2^)	Underweight	96 (18.0)	26 (19.8)	6.255	0.100
	Normal	225 (42.2)	48 (36.6)		
	Overweight	93 (17.4)	16 (12.2)		
	Obese	119 (22.3)	41 (31.3)		
WHtR	<0.50	436 (81.8)	99 (75.6)	2.606	0.106
	>0.50	97 (18.2)	32 (24.4)		
% Body Fat	<30%	291 (54.6)	59 (45.0)	3.854	0.05

Although mean BMI appeared to be in the normal range for Asian Indians, the distribution showed that in both categories of HbA1c i.e. < 5.7% and 5.7-6.4%, a considerable percentage of subjects were either overweight or obese, the percentage of obese subjects being higher in the prediabetic group with HbA1c between 5.7%-6.4%, while the percentage with normal BMI was lower (**Table 2**). A higher percentage of prediabetics were obese (31.3%), or had WHtR >0.5 (24.4%) or higher percent body fat (55.0%) as compared to those with HbA1c < 5.7%, although there was no significant difference between the two groups (**Table 2**). However, the percentage of prediabetics with percent body fat exceeding 30% was significantly higher.

Mean fasting blood glucose and 2-hour post glucose levels were significantly higher in the prediabetic group than in those with normal HbA1c. Fasting and stimulated insulin also tended to be higher for the prediabetic group, but the difference between them and those with normal HbA1c levels was not statistically significant (**Table 3**).

**Table 3:** Comparison of blood glucose and insulin in persons with normal HbA1c (<5.7) and prediabetics (HbA1c: 5.7-6.4).

Fasting and Stimulated Blood Glucose and Insulin		HbA1c <5.7 (n=533) n (%)	HbA1c 5.7-6.4 (n=131) n (%)	t value	P
Mean ±SD					
**Fasting Blood Glucose (mg/dL)**		82.0±7.7	83.4±7.7	-1.895	0.059
**2 -hour Glucose (mg/dL)**		94.1±17.3	99.7±22.9	-3.115	0.002
**Fasting Insulin (mIU/L)**		8.9±4.6	9.7±4.8	-1.794	0.073
**Stimulated** **Insulin at 2 hours (mIU/L)**		79.6 ±61.5	82.5±63.1	-0.480	0.632
Number (%) subjects with normal and higher blood glucose and Insulin				Chi sq	P
**Fasting Blood Glucose**	<100 mg/dl	525(98.5)	131(100)	1.990	0.158
	≥100mg/dl	8(1.5)	0(0)		
**2 hour Glucose**	<140mg/dl	525(98.5)	125(95.4)	4.831	0.028
	≥140mg/dl	8(1.5)	6(4.6)		
**Fasting Insulin**	<15 mIU/L	477(89.5)	119(90.8)	0.207	0.649
	≥15mIU/L	56(10.5)	12(9.2)		
**Stimulated** **Insulin**	<80 mIU/L	323(60.6)	81(61.8)	0.067	0.796
	≥80mIU/L	210(39.4)	50(38.2)		

Blood glucose levels were elevated in 16 of the 664 participants (2.4%), although their HbA1c levels were normal (elevated fasting glucose n=8 and 2 hour elevated stimulated glucose n=8). Similarly, 68 participants had fasting insulin>15 mIU/L. Among these, 56 participants had HbA1c less than 5.7. For almost two-fifths of the participants (39.4%, n=260), the stimulated insulin was ≥80 mIU/L, although the HbA1c for 210 persons was <5.7% (**Table 3**).

Further, among the eight persons (1.5%) whose fasting glucose was above 100mg/dl, five had HbA1c between 5.0%5.4% and three had between 5.5% and 5.6%. It was observed that a substantial proportion of persons whose HbA1c levels were in the normal range, had elevated 2-hour stimulated insulin levels.

**Figure 1:**
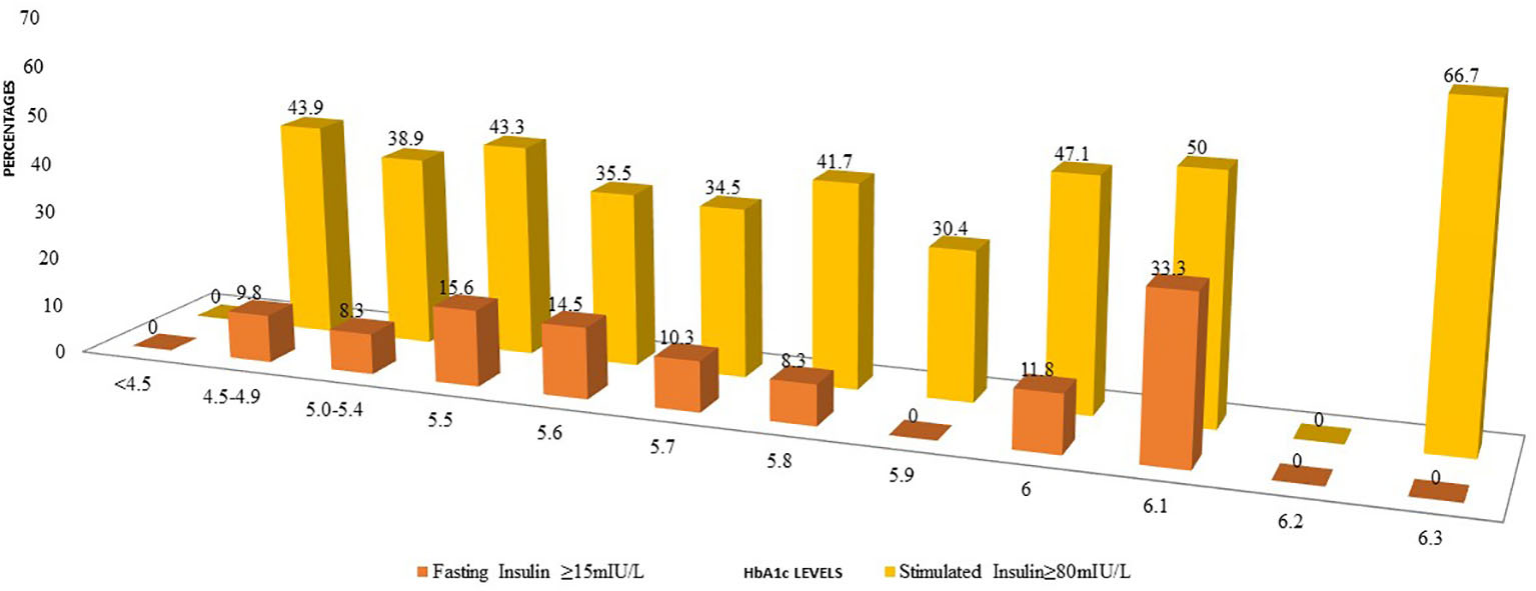
Comparison of percentages of participants with elevated fasting insulin or post 2-H stimulated insulin at diffrent levels of HbA1c.

Even among those with HbA1c between 4.5%-4.9%, 43.9% of participants had higher 2-hour stimulated insulin levels (**Figure 1**). A similar trend was observed for fasting insulin although the percentages were less than the percentages with high stimulated insulin.

BMI and body composition measurements were also compared between those with normal HbA1c < 5.7% and HbA1c between 5.7% and 6.4%. In each of these groups, comparisons were made between those with normal and elevated fasting insulin. In the group with normal HbA1c, mean values for BMI, WC, WHtR, percent body fat as well as visceral fat and muscle mass were significantly higher when their fasting insulin was high as compared to those with normal fasting insulin. Among those with elevated HbA1c, a similar trend was seen with those whose fasting insulin≥15m IU/L. The mean values for all anthropometric indices, percent body fat and abdominal fat were higher for this except for muscle mass (**Table 4**). A similar trend was seen for those with normal HbA1c but 2-hour post glucose stimulated insulin was above 80 m IU/L and in the group whose HbA1c was between 5.7% and 6.4%.

**Table 4:** Comparison of mean anthropometric indices between participants having HbA1c<5.7 and those with HbA1c between 5.7 and 6.4 with normal and elevated fasting and stimulated insulin levels.

	HbA1C<5.7 Mean ±SD (Median)		t p	HbA1c 5.7-6.4 Mean ±SD (Median)		T p
	Fasting Insulin < 15m IU/L (n=477)	Fasting Insulin ≥ 15m IU/L (n=56)		Stimulated Insulin < 15m IU/L (n=119)	Stimulated Insulin ≥ 15m IU/L (n=12)	
**BMI** **(kg/m^2^)**	22.0±4.1 (21.3)	25.7±4.6 (25.4)	-6.295 0.000	22.6±5.2 (21.9)	28.1±4.8 (28.4)	-3.532 0.001
**WC** **(cm)**	70.4±9.7 (69.0)	77.7±9.3 (77.0)	-5.382 0.000	71.0±10.5 (70.0)	79.6±10.4 (78.0)	-2.704 0.008
**WHR**	0.76±0.06 (0.76)	0.78±0.05 (0.78)	-2.367 0.018	0.76±0.0 (0.75)	0.76±0.05 (0.75)	0.005 0.996
**WHtR**	0.44±0.06 (0.43)	0.49±0.05 (0.49)	-5.512 0.000	0.45±0.06 (0.44)	0.51±0.07 (0.49)	-3.162 0.002
**%Body** **Fat**	27.5±7.9 (28.0)	34.2±7.8 (34.5)	-6.005 0.000	29.6±8.1 (30.0)	37.7±5.2 (39.0)	-3.423 0.001
**Visceral** **Fat**	4.0±2.8 (3.0)	6.1±3.1 (6.0)	-5.224 0.000	4.3±3.1 (4.0)	6.4±2.2 (7.0)	-2.225 0.028
**Muscle** **Mass** **(kg)**	37.5±8.0 (36.0)	39.7±8.4 (38.0)	-1.926 0.000	37.02±7.2 (36.0)	37.3±6.3 (39.5)	-0.147 0.883

	Stimulated Insulin< 80 m IU/L (n=323)	Stimulated Insulin≥ 80 m IU/L (n=210)	t p	Stimulated Insulin< 80 m IU/L (n=81)	Stimulated Insulin≥ 80 m IU/L (n=50)	T p
**BMI** **(kg/m^2^)**	22.0±4.2 (21.1)	22.9±4.4 (22.5)	-2.240 0.025	22.5±5.6 (21.7)	24.3±4.8 (24.4)	-1.869 0.064
**WC** **(cm)**	70.2±9.7 (68.0)	72.6±10.0 (72.0)	-2.754 0.006	69.9±11.2 (68.0)	74.7±9.3 (73.5)	-2.503 0.014
**WHR**	0.75±0.06 (0.75)	0.78±0.06 (0.77)	-4.964 0.000	0.75±0.05 (0.74)	0.78±0.06 (0.75)	-3.044 0.003
**WHtR**	0.44±0.06 (0.43)	0.46±0.07 (0.45)	-3.444 0.001	0.44±0.07 (0.43)	0.48±0.05 (0.47)	-3.085 0.002
**%Body** **Fat**	27.2±8.3 (27.0)	29.6±7.7 (30.0)	-3.286 0.001	28.6±8.4 (29.0)	33.2±7.0 (34.0)	-3.204 0.002
**Visceral** **Fat**	4.0±2.9 (3.00	4.5±3.0 (4.0)	-2.028 0.043	4.2±3.2 (3.0)	5.0±2.8 (5.0)	-1.488 0.139
**Muscle** **Mass** **(kg)**	38.3±8.3 (37.0)	36.8±7.5 (35.0)	-2.088 0.037	37.0±7.6 (36.0)	37.1±6.1 (36.5)	-0.020 0.984

## 4 Discussion

In the present study we measured fasting blood glucose, 2 h- glucose stimulated blood glucose and HbA1c to identify those who were prediabetic (9, 12, 29). We also measured fasting insulin and 2- h glucose stimulated insulin in the same subjects. We determined how many individuals having normal HbA1c and those who were prediabetic, were hyperinsulinemic. Our findings showed that as per the ADA criteria for blood glucose only 4.2% were prediabetic whereas 19.9% had HbA1c values in prediabetic range (5.7% - 6.4%). That almost one-fifth of the young adults were prediabetic, highlights the need to pay attention to this issue.

Further, it is indeed of concern that almost 40% of the participants had hyperinsulinemia based on the 2-hour post glucose stimulated insulin levels. Also, about 22.5% of these participants had first degree relatives with diabetes which would increase their risk of developing diabetes as they grow older. Early detection and intervention would be mandatory specifically in this high-risk group that would help to alleviate the serious health complications that may exist in association with hyperinsulinemia (22, 23).

The findings of the present study are in contrast to an earlier study wherein, insulin deficiency was reported as the ‘major driver’ in young Indians unlike in young European individuals in whom obesity and insulin resistance predominate (34). However, these conclusions were based on data of young adults who were already diagnosed with diabetes, whereas our participants were apparently healthy. adolescents and young adults. In the present study, three young adults were detected as diabetic but they were unaware about it at the time of screening. Being a known diabetic was one of the exclusion criteria in our study.

Adolescents and young adults are vulnerable because they are more likely to have erratic lifestyles and poor food habits increasing the risk of hyperinsulinemia. A very early study by some members of our group showed that among 65 patients with identified polycystic ovarian syndrome, 71 percent were insulin resistant (32). A study on 778 adolescent and young girls mostly from lower socioeconomic strata in Mumbai showed that 19.2% of diagnosed PCOS cases were hyperinsulinemic (serum insulin >15 μlU/mL) (35). Also, the prevalence of overweight/obesity were quite high and 20.7% of the girls were obese. Dysregulated insulin secretion and/or clearance resulting in chronically elevated insulin or hyperinsulinemia, without hypoglycemia is common in obesity and metabolic disorders (36). In subjects with obesity but without diabetes or hypertension, hyperinsulinemia and insulin hypersecretion precede insulin resistance.

Cohort studies have shown that different subjects with similar degrees of insulin sensitivity may exhibit a range of insulin secretion. In the Relationship between Insulin Sensitivity and Cardiovascular Disease (RISC) study (37), individuals with insulin hypersecretion tended to be older, had higher percent fat mass, worse lipid profile and higher liver insulin resistance indices compared with the rest of the cohort. In the RISC study, preexposure to hyperinsulinemia stimulated a greater insulin-induced secretory response independently of insulin sensitivity. Hence, hyperinsulinemia is self-perpetuating and is more likely to be a primary defect rather than a compensation for insulin resistance in the general population. A study in 2021 suggested that a reverse order and place hyperinsulinemia mechanistically upstream of insulin resistance’ (38). Hyperinsulinemia is probably primary and is more likely to be a cause rather than a consequence of insulin resistance (39), suggesting that insulin resistance is the body’s defense mechanism to protect important vital tissues from the metabolic stress of hypoglycemia. Further, GLUT 4 expressions get severely disrupted contributing to insulin resistance. There may be defective intracellular signalling of GLUT 4 translocation from the intracellular compartment to the plasma membrane (40). Majority of the young adults in the present study had either a first degree and/or a second degree relative who were diabetic. Since Type 2 diabetes is heritable (40), it is possible that in young Indian adults there could be impairment in the skeletal muscle cells and adipocytes.

Chronic inflammation associated with obesity is of concern as excess body fat, particularly abdominal adipose tissue influence insulin resistance and increases risk of type 2 diabetes (30). In insulin-resistant states, inflammatory markers like tumor necrosis factor-α, interleukin-6, C-reactive protein are elevated (41). In obesity there is immune dysregulation, that leads to chronic low-grade inflammation (42). Consequently, early intervention when hyperinsulinemia is detected is critical to prevent the chronic and degenerative NCDs (38).

In the present study, only 3% (n=16) of the participants had fasting or 2-hour sugar levels above the cut-off (n=16), but their HbA1c levels were normal. Among the 68 participants whose fasting insulin levels were >15 mIU/L, 56 participants had HbA1c <5.7%. Further among 260 participants with stimulated insulin levels ≥80 mIU/L, 210 participants HbA1c<5.7%. Clearly, a considerably high percentage (39.3%) were hyperinsulinemic. This raises the question; would it be prudent to use hyperinsulinemia to identify persons who need intervention to prevent progression to type 2DM, in clinical settings. Our findings indicated that among those with higher levels of fasting and stimulated insulin levels (but normal HbA1c), mean BMI was significantly higher than those who had normal insulin levels. A similar trend was observed among those who could be designated as prediabetic based on the HbA1c levels. Another question that arises is do we need to revisit the cut off of HbA1c for South Asians particularly the overweight and obese phenotypes? While obesity is generally identified using BMI, it is well known that this does not reflect that body fat is not homogeneously distributed and also does not help in distinguishing those who are obese but metabolically healthy versus those who may be normal yet metabolically unhealthy (43). Visceral fat is associated with insulin resistance as well as chronic inflammation that in turn is linked to metabolic syndrome (43, 44). Asians have a relatively higher body fat content for the same or lower body mass index in contrast to Caucasians, as they are probably more likely to accumulate visceral fat and have greater chances of abdominal adiposity (45, 46). In our study, this group with normal HbA1c yet higher fasting or stimulated insulin had higher BMI and percent body fat based on both higher-than-normal levels of fasting as well as 2-hour stimulated insulin.

An International Expert Committee has proposed that if HbA1c levels are above 6.5%, then a diagnosisof diabetes can be made but should be confirmed with a repeat HbA1c test, unless clinical symptomsor glucose levels are above 200 mg/dl (11.1 mmol/l) (47). However, this decision was based on cross-sectional data on the relationship between HbA1c and risk of future complications (retinopathy) in Western populations. Our data from an Asian Indian population indicates that the HbA1c cut off point appropriate for diagnosing diabetes may be different for non-western populations. Our data suggest that at HbA1c<5.7% in Asian Indians, few pre-diabetics with fasting glucose and 2-hour glucose were missed, but a large percentage had hyperinsulinemia at an HbA1c value that is considered normal. Our data highlights that reliance on HbA1c alone would lead to missing out many hyperinsulinemic individuals, which is of concern, given the strong evidence that lifestyle management of those with IGT can reduce the rate of progression to diabetes.

Admittedly, the HbA1c test has advantages, it can be measured at any time of the day with a small sample of blood and it does not require the cumbersome glucose load test. However, our data suggest that it may be worthwhile to further check the insulin levels of those who have a higher BMI or percent body fat and visceral/abdominal adiposity, even if their HbA1c is below 5.7%. It has been suggested that “the insulin assay, measuring both fasting and after an OGTT, seems to be the earliest biomarker for diagnosing T2D” (48). The need to include hyperinsulinemia along with high BMI or percent body fat (even if HbA1c is normal) as one of the factors to identify pre diabetes and prevent its progression into frank diabetes is the need of the day for the higher risk group.

Hyperinsulinaemia in the absence of impaired glucose tolerance and normal HbA1c may provide a much earlier indicator of detection for risk of metabolic disease and progression to metabolic syndrome and diabetes mellitus, which affects millions in India. For community screening HbA1c would be still a preferred marker for the diagnosis of prediabetes. However, for individuals at high risks like obesity, central adiposity, family history of diabetes or those who symptomatic (acanthosis nigricans, PCOS) should be tested for hyperinsulinemia and insulin resistance.

## Data availability statement

The original contributions presented in the study are included in the article/Supplementary Material. Further inquiries can be directed to the corresponding author.

## Ethics statement

The studies involving human participants were reviewed and approved by Intersystem Biomedical Ethics Committee, Mumbai, India (ISBEC version 2 dated 12th August, 2017). Written informed consent to participate in this study was provided by the participants and for for the participants 16-18 years old was provided by legal guardian/next of kin.

## Author contributions

RV hypothesis was constructed by her and has contributed to writing of the paper. JM- She was the principal investigator and has contributed in finalization of the paper. SU- She has contributed in statistical analysis and writing the paper. SD- She has contributed in management of data and writing of the paper. PM, SS, RB- They have contributed in recruiting the participants and management of the study. AM- She was the supervising physician. SK- He was the Co-PI and has contributed in finalization of the paper. AV- He was the mentor and has contributed in finalization of the paper. All authors contributed to the article and approved the submitted version

## References

[1] International Diabetes Federation. Prevalence of diabetes (20–79 years), 2019, in: Diabetes atlas (2019). Available at: https://www.diabetesatlas.org/upload/resources/material/20191218_144626_sea_factsheet_en.pdf (Accessed 25 April 2022).

[2] Anjana RM Mohan D Rajendra P Mahanta J Narain K Das HK. Prevalence of diabetes and prediabetes in 15 states of India: results from the ICMR–INDIAB population-based cross-sectional study. Lancet Diabetes Endocrinol. (2017) 5(8):585–96. doi: 10.1016/S2213-8587(17)30174-2 28601585

[3] National family health survey-5 (2019-2020). Available at: http://rchiips.org/nfhs/NFHS-5_FCTS/India.pdf.

[4] Heianza Y Hara S Arase Y Saito K Fujiwara K Tsuji H. HbA1c 5·7-6·4% and impaired fasting plasma glucose for diagnosis of prediabetes and risk of progression to diabetes in Japan 351 (TOPICS 3): a longitudinal cohort study. Lancet. (2011) 378(9786):147–55. doi: 10.1016/S0140-3526736(11)60472-8 21705064

[5] Richter B Hemmingsen B Metzendorf M-I Takwoingi Y. Development of type 2 diabetes mellitus in people with intermediate hyperglycaemia. Cochrane Database Syst. Rev. (2018) 10:CD012661. doi: 10.1002/14651858.CD012661 30371961 PMC6516891

[6] Tabak AG Herder C Rathmann W Brunner EJ Kivimäki M. Prediabetes: a high-risk state for 357 diabetes development. Lancet. (2012) 379(9833):2279–90. doi: 10.1016/S0140-6736(12)60283-9 PMC3891203 22683128

[7] Huang Y Cai X Mai W Li M Hu Y. Association between prediabetes and risk of cardiovascular disease and all-cause mortality: systematic review and meta-analysis. BMJ. (2016) 355:i5953. doi: 10.1136/bmj.i5953 27881363 PMC5121106

[8] Yeboah J Bertoni AG Herrington DM Post WS Burke GL. Impaired fasting glucose and the risk of incident diabetes mellitus and cardiovascular events in an adult population: MESA (Multi-ethnic study of atherosclerosis). J. Am. Coll. Cardiol. (2011) 58(2):140–6. doi: 10.1016/j.jacc.2011.03.025 PMC3146297 21718910

[9] American Diabetes Association. 2. Classification and diagnosis of diabetes: standards of medical care in diabetes-2018. Diabetes Care (2018) 41(Suppl 1):S13–28. doi: 10.2337/dc19-S002 29222373

[10] Beulens J Rutters F Rydén L Schnell O Mellbin L Hart HE. Risk and management of pre-diabetes. Eur. J. Prev. Cardiol. (2019) 26(2_suppl):47–54. doi: 10.1177/2047487319880041 31766914

[11] Cai X Zhang Y Li M Wu JH Mai L Li J. Association between prediabetes and risk of all cause mortality and cardiovascular disease: updated meta-analysis. BMJ (2020) 370:m2297. doi: 10.1136/bmj.m2297 32669282 PMC7362233

[12] World Health Organization. Definition, diagnosis and classification of diabetes mellitus and its complications report of world health organization consultation. In: Part 1: diagnosis and classification of diabetes mellitus. Geneva: WHO (1999).

[13] American Diabetes Association Professional Practice Committee. 2. classification and diagnosis of diabetes: standards of medical care in diabetes-2022. Diabetes Care (2022) 45(1):S17–38. doi: 10.2337/dc22-S002 34964875

[14] Cosentino F Grant PJ Aboyans V Bailey CJ Ceriello A Delgado V. 2019 ESC guidelines on diabetes, pre- diabetes, and cardiovascular diseases developed in collaboration with the EASD: the task force for diabetes, pre-diabetes, and cardiovascular diseases of the European society of cardiology (ESC) and the European association for the study of diabetes (EASD). Eur. Heart J. (2020) 41(2):255–323. doi: 10.1093/eurheartj/ehz486 31497854

[15] Mohan V Vijayachandrika V Gokulakrishnan K Anjana RM Ganesan A Weber MB. A1C cut points to define various glucose intolerance groups in Asian indians. Diabetes Care (2010) 33(3):515–9. doi: 10.2337/dc09-1694 PMC2827500 19903752

[16] Thewjitcharoen Y Jones Elizabeth A Butadej S Nakasatien S Chotwanvirat P Wanothayaroj E. Performance of HbA1c versus oral glucose tolerance test (OGTT) as a screening tool to diagnose dysglycemic status in high-risk Thai patients. BMC Endocr. Disord. (2019) 19(1):23. doi: 10.1186/s12902-019-0339-6 30770743 PMC6377733

[17] Kim JH Kim GW Lee MY Shin JY Shin YG Koh SB. Role of HbA1c in the screening of diabetes mellitus in a Korean rural community. Diabetes Metab. J. (2012) 36(1):37–42. doi: 10.4093/dmj.2012.36.1.37 22363920 PMC3283825

[18] Florkowski C. HbA1c as a diagnostic test for diabetes mellitus - reviewing the evidence. Clin. Biochem. Rev. (2013) 34(2):75–83. 24151343 PMC3799221

[19] Nair M Prabhakaran D Narayan KM Sinha R Lakshmy R Devasenapathy N. HbA(1c) values for defining diabetes and impaired fasting glucose in Asian indians. Prim Care Diabetes. (2011) 5(2):95–102. doi: 10.1016/j.pcd.2011.02.002 21474403 PMC3117965

[20] Radhakrishna P Vinod KV Sujiv A Swaminathan RP. Comparison of hemoglobin A1c with fasting and 2-h plasma glucose tests for diagnosis of diabetes and prediabetes among high-risk south indians. Indian J. Endocrinol. Metab. (2018) 22:50–6. doi: 10.4103/ijem.IJEM_254_17 PMC5838911 29535937

[21] Herman WH Cohen RM. Racial and ethnic differences in the relationship between HbA1c and blood glucose: implications for the diagnosis of diabetes. J. Clin. Endocrinol. Metab. (2012) 97(4):1067–72. doi: 10.1210/jc.2011-1894 PMC3319188 22238408

[22] Petersen MC Shulman GI. Mechanisms of insulin action and insulin resistance. Physiol. Rev. (2018) 98(4):2133–223. doi: 10.1152/physrev.00063.2017 PMC6170977 30067154

[23] Thomas DD Corkey BE Istfan NW Apovian CM. Hyperinsulinemia: an early indicator of metabolic dysfunction. J. Endocr. Soc (2019) 3(9):1727–47. doi: 10.1210/js.2019-00065 PMC6735759 31528832

[24] Ferrannini E Natali A Bell P Cavallo-Perin P Lalic N Mingrone G. Insulin resistance and hypersecretion in obesity. Eur. Group Study Insulin Resistance (EGIR). J. Clin. Invest. (1997) 100(5):1166–73. doi: 10.1172/JCI119628 PMC508292 9303923

[25] Kraft JR. Detection ofdiabetes mellitus *in situ* (occult diabetes). Lab. Med. (1975) 6:10–22. doi: 10.1093/labmed/6.2.10

[26] Crofts C Schofield G Zinn C Wheldon M Kraft J. Identifying hyperinsulinaemia in the absence of impaired glucose tolerance: an examination of the kraft database. Diabetes Res Clin Pract (2016) 118:50–7. doi: 10.1016/j.diabres.2016.06.007 27344544

[27] Wells JC Pomeroy E Walimbe SR Popkin BM Yajnik CS. The elevated susceptibility to diabetes in India: an evolutionary perspective. Front. Public Health (2016) 4:145. doi: 10.3389/fpubh.2016.00145 27458578 PMC4935697

[28] Misra A Ramachandran A Saboo B Kesavadev J Sosale A Joshi S. Screening for diabetes in India should be initiated at 25 years age. Diabetes Metab. Syndr Clin Res Rev (2021) 14(6):102321. doi: 10.1016/j.dsx.2021.102321 34739907

[29] Standards of Medical Care in Diabetes. Summary of Revisions. Diabetes Care (2017) 40(Supplement_1):S4-S5. doi: 10.2337/dc17-S003 27979887

[30] Madan J Desai S Moitra P Salis S Agashe S Battalwar R. Effect of almond consumption on metabolic risk factors-glucose metabolism, hyperinsulinemia, selected markers of inflammation: a randomized controlled trial in adolescents and young adults. Front. Nutr. (2021) 8:668622. doi: 10.3389/fnut.2021.668622 34249987 PMC8264510

[31] Ashwell M Gibson S. A proposal for a primary screening tool: 'Keep your waist circumference to less than half your height'. BMC Med. (2014) 12:207. doi: 10.1186/s12916-014-0207-1 25377944 PMC4223160

[32] Shringi M Vaidya RA Vaidya AB. Insulin resistance in polycystic ovarian syndrome: a study of 90 patients. J. Endocrinol. Metab. (2003) 1:19–23.

[33] Lee S Choi S Kim HJ Chung YS Lee KW Lee HC. Cut off values of surrogate measures of insulin resistance for metabolic syndrome in Korean non-diabetic adults. J. Korean Med. Sci. (2006) 21(4):695–700. doi: 10.3346/jkms.2006.21.4.695 16891815 PMC2729893

[34] Prasad RB Asplund O Shukla SR Wagh R Kunte P Bhat D. Subgroups of patients with young-onset type 2 diabetes in India reveal insulin deficiency as a major driver. Diabetologia. (2022) 65:65–78. doi: 10.1007/s00125-021-05543-y 34689214 PMC8660725

[35] Joshi B Mukherjee S Patil A Purandare A Chauhan S Vaidya R. A cross-sectional study of polycystic ovarian syndrome among adolescent and young girls in Mumbai, India. Indian J. Endocrinol. Metab. (2014) 18(3):317–24. doi: 10.4103/2230-8210.131162 PMC4056129 24944925

[36] Reaven GM. Banting lecture 1988. role of insulin resistance in human disease. Diabetes (1988) 37(12):1595–607. doi: 10.2337/diab.37.12.1595 3056758

[37] de Rooij SR Nijpels G Nilsson PM Nolan JJ Gabriel R Bobbioni-Harsch E. Relationship between insulin sensitivity and cardiovascular disease (RISC) investigators. low-grade chronic inflammation in the relationship between insulin sensitivity and cardiovascular disease (RISC) population: associations with insulin resistance and cardiometabolic risk profile. Diabetes Care (2009) 32(7):1295–301. doi: 10.2337/dc08-1795 PMC2699728 19366964

[38] Janssen J.A.M.J.L. Hyperinsulinemia and its pivotal role in aging, obesity, type 2 diabetes, cardiovascular disease and cancer. Int. J. Mol. Sci. (2021) 22:7797. doi: 10.3390/ijms22157797 34360563 PMC8345990

[39] Corkey BE. Banting lecture 2011: hyperinsulinemia: cause or consequence? Diabetes. (2012) 61:4–13. doi: 10.2337/db11-1483 22187369 PMC3237642

[40] Vargas E Podder V Sepulveda MAC. Physiology, glucose transporter type 4. Stat. Pearls (2022) 3(3):141–6.

[41] De Luca C Olefsky JM. Inflammation and insulin resistance. FEBS Lett. (2008) 582(1):97–105. doi: 10.1016/j.febslet.2007.11.057 18053812 PMC2246086

[42] Zatterale F Longo M Naderi J Raciti GA Desiderio A Miele C. Chronic adipose tissue inflammation linking obesity to insulin resistance and type 2 diabetes. Front. Physiol. (2020) 10:1607. doi: 10.3389/fphys.2019.01607 32063863 PMC7000657

[43] Hwang YC Kahn SE Leonetti DL McNeely MJ. Visceral abdominal fat accumulation predicts the conversion of metabolically healthy obese subjects to an unhealthy phenotype. Int. J. Obes. (Lond) (2015) 39:1365–70. doi: 10.1038/ijo.2015.75 PMC4564328 25920773

[44] Després JP Nadeau A Tremblay A Ferland M Moorjani S Lupien PJ. Role of deep abdominal fat in the association between regional adipose tissue distribution and glucose tolerance in obese women. Diabetes. (1989) 38(3):304–9. doi: 10.2337/diab.38.3.304 2645187

[45] Deurenberg P Deurenberg-Yap M Guricci S. Asians are different from caucasians and from each other in their body mass index/body fat per cent relationship. Obes. Rev. (2002) 3(3):141–6. doi: 10.1046/j.1467-789X.2002.00065.x 12164465

[46] Camhi SM Bray GA Bouchard C Greenway FL Johnson WD Newton RL. The relationship of waist circumference and BMI to visceral, subcutaneous, and total body fat: sex and race differences. Obes. (Silver Spring). (2011) 19(2):402–8. doi: 10.1038/oby.2010.248 PMC3960785 20948514

[47] International Expert Committee. International expert committee report on the role of the A1C assay in the diagnosis of diabetes. Diabetes Care (2009) 32(7):1327–34. doi: 10.2337/dc09-9033 PMC2699715 19502545

[48] DiNicolantonio JJ Bhutani J OKeefe JH Crofts C. Postprandial insulin assay as the earliest biomarker for diagnosing pre-diabetes, type 2 diabetes and increased cardiovascular risk. Open Heart (2017) 4(2):e000656. doi: 10.1136/openhrt-2017-000656 29225902 PMC5708305

